# Immune checkpoint inhibitor sintilimab-induced lethal myocarditis overlapping with myasthenia gravis in thymoma patient: A case report

**DOI:** 10.1097/MD.0000000000033550

**Published:** 2023-04-14

**Authors:** Chen Wang, Bingdi Zhong, Jing He, Xiaohong Liao

**Affiliations:** a Department of Oncology, Ganzhou People’s Hospital, Ganzhou, Jiangxi Province, China.

**Keywords:** case report, immune checkpoint inhibitor, myocarditis, sintilimab, thymoma

## Abstract

Immune checkpoint inhibitors have been extensively used and significantly improved the clinical outcomes in multiple types of cancer. But the immune-related adverse events occur frequently, particularly in thymoma. The cardiac immune-related adverse, which is relatively rare but fatal, have been increasing reported.

**Patient concerns:**

A 45-year-old thymoma patient was admitted to our hospital after receiving anti-programmed cell death-1 treatment with sintilimab 14 days later, accompanied by abdominal pain, intermittent chest tightness and dizziness.

**Diagnoses:**

The laboratory tests revealed elevated serum troponin I. Electrocardiogram reported the prolongation of QTc interval. Echocardiography showed small amount of pericardial effusion, a left ventricular ejection fraction of 71%. Coronary artery computed tomography angiography revealed localized noncalcified plaque in the middle of the left anterior descending artery and mild stenosis of the lumen. Enhanced computed tomography scanning of the whole abdomen showed no abnormal signs in the parenchyma organs. Combining the results of the examinations, the Immune checkpoint inhibitor induced myocarditis was diagnosed.

**Interventions:**

The patient was treated with glucocorticoids (120 mg/day, IV, methylprednisolone) within 24 hours of admission. Seven days later, the patient experienced tachy ventricular arrhythmia and cardiogenic shock and was transferred to intensive care unit after electrical cardioversion, tracheal intubation and cardiopulmonary resuscitation. Intravenous immunoglobulin therapy at 25 g/day was given and methylprednisolone was reduced to 40 mg/day for the next 3 days. Intravenous esmolol and lidocaine were used for correcting arrhythmias. Ventilator positive pressure ventilation was used for respiratory support. She was administrated with plasmapheresis when the electrocardiogram monitoring showed ventricular arrhythmia storms.

**Outcome:**

The patient progressed to ventricular arrhythmia storms and cardiac failure, which eventually resulted in death.

**Lessons:**

The case aims to raise awareness of immune-mediated cardiotoxicity and bring thoughts to the prospects of immunotherapy in thymoma.

## 1. Introduction

Thymic epithelial tumors (TETs) are the most common anterior mediastinal tumors in adults.^[1]^ there are 1.3 to 3.2 cases per million TETs per year, and has been a gradual increasing in recent years.^[1]^ TETs have been classified into types A, AB, B1, B2, B3, and thymic carcinoma according to the morphology, malignancy and ratio of thymic epithelial cells to lymphocytes.^[1]^ At present, surgery followed by chemotherapy or radiotherapy is the main treatment for localized TETs, and chemotherapy, chemoradiotherapy or targeted therapy is recommended for the advanced and metastatic patients.^[2]^ However, the efficacy of available treatment options is limited for these unresectable TETs patients.^[2]^ In recent years, immune checkpoint inhibitors (ICIs) to target cytotoxic T-lymphocyte activator-4, programmed cell death-1 (PD-1) and programmed cell death ligand-1 (PD-L1) showed great efficacy in antitumor therapy.^[3]^ Several researches have explored the high expression of PD-L1 on thymic tumor cells and abundant CD8 + lymphocytes, which provides a strong rational for implementing ICIs which target PD-1/PD-L1 in the treatment of TETs.^[4]^ However, immune-related adverse events (irAEs) including endocrinopathies, myositis, and pneumonitis, occurred more frequently than other cancers.^[3,5]^

Although myasthenia gravis (MG) is reported to be one of the most common irAE in TET patients administrated with ICIs, the most serious irAE is myocarditis due to the high mortality rate.^[6]^ Sintilimab, a fully recombinant human anti-PD-1 IgG4 monoclonal antibody, which can bind to PD-1 and then inhibit the binding of PD-1 receptors on T cells to PD-L1 on tumor cells, finally activate cytotoxic T lymphocytes and improve their antitumor activity. The adverse events induced by sintilimab include, but are not limited to, pneumonitis, hepatitis, endocrinopathies, colitis and myocarditis.^[7]^ Here we report a case of ICI-induced lethal myocarditis overlapping with MG secondary to sintilimab therapy for a thymoma patient. We present the following case in accordance with the CARE reporting checklist.

## 2. Case presentation

**Figure 1.**
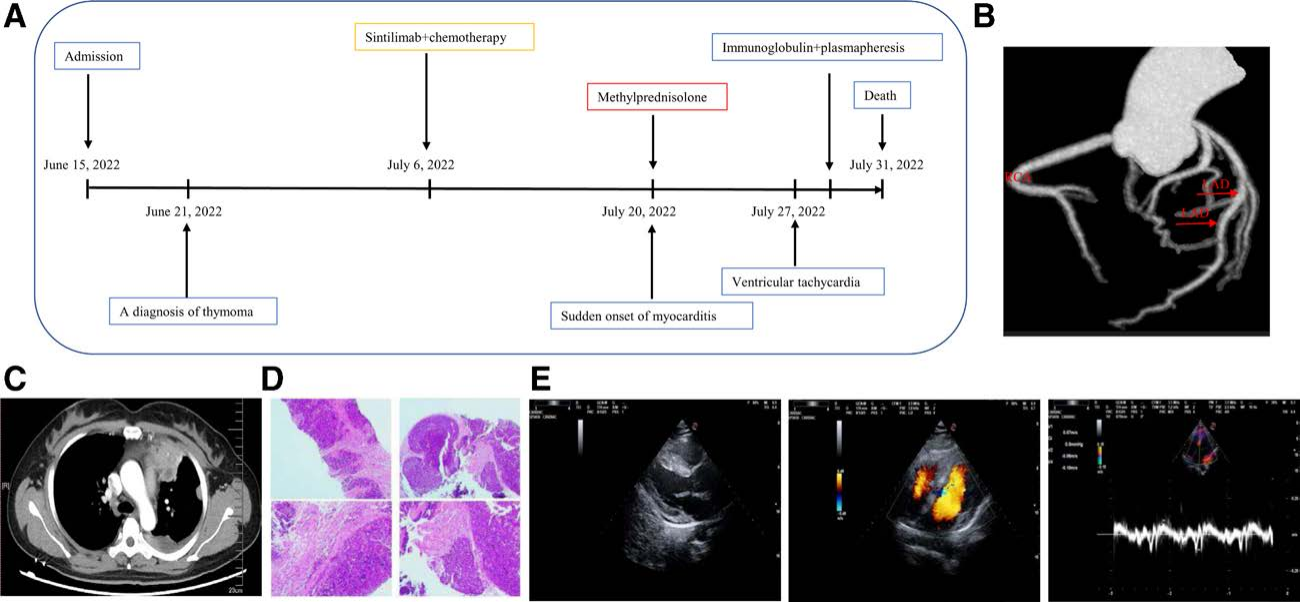
Case presentation. Timeline of the disease diagnosis and treatment, (A) the Coronary artery CT angiography (CTA) imaging, (B), the enhanced chest computed tomography (CT), (C) the autopsy of the para-mediastinal mass, (D) the echocardiography imaging, and (E). RCA: right coronary artery; LAD: left anterior descending artery.

A 45-year-old Chinese woman was admitted to Ganzhou people’s hospital presenting with a history of pain in left anterior chest area for more than 2 years and aggravated pain for more than half a month on June 15, 2022. Chest computed tomography (CT) revealed a para-mediastinal mass (Fig. 1C). Enlarged lymph nodes and multiple left pleural masses were considered to be metastatic tumors. She underwent biopsy of the para-mediastinal mass and was diagnosed as thymoma, type B2 (Fig. 1D). Her family history and social history were noncontributory. Then she was treated with 1 cycle of chemotherapy (nab-paclitaxel, 400 mg, day1 and carboplatin, 600 mg, day1) combined with sintilimab (200 mg, day1) on July 6, 2022, in Guangdong Provincial People’s Hospital.

**Figure 2.**
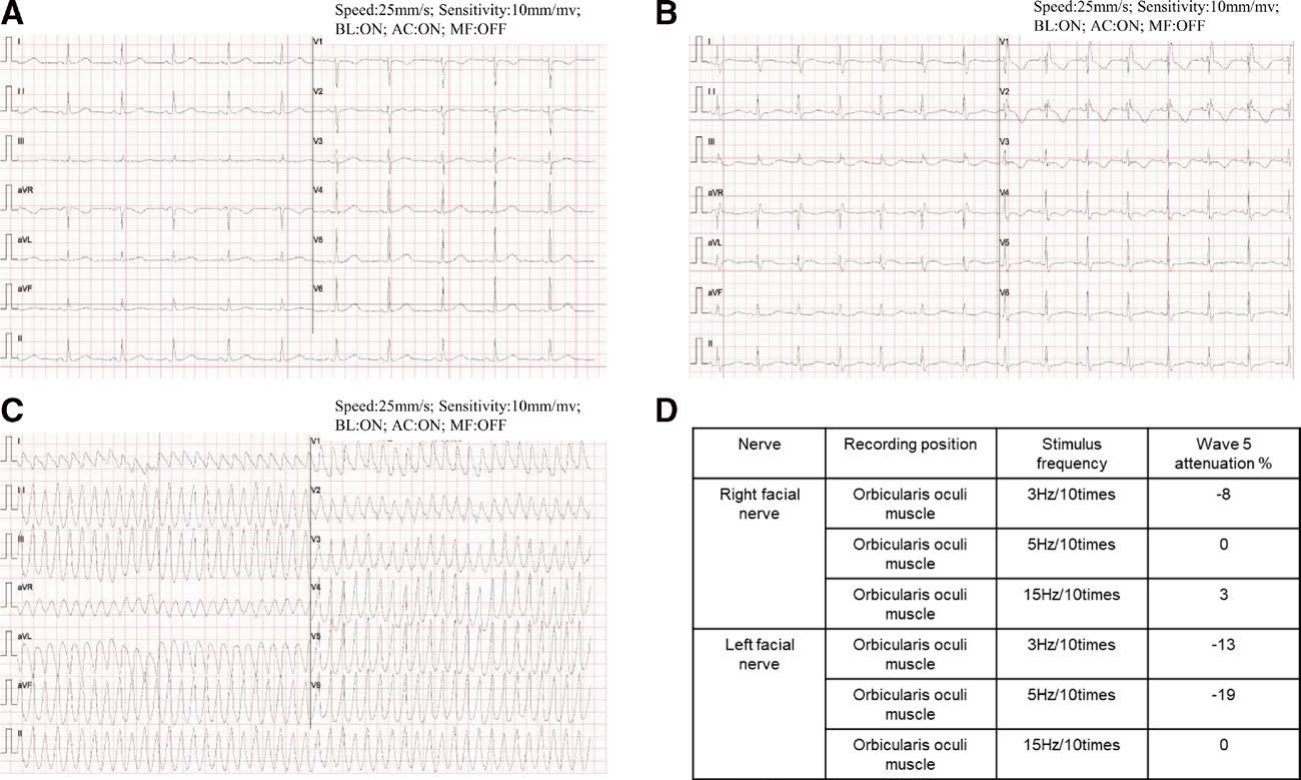
Electrocardiogram changes in the process of the disease. The prolongation of QTc interval, (A) complete right bundle branch block, the prolongation of QTc interval and the change of T wave, (B) the ventricular tachycardia, and (C) and the electromyogram (EMG) results showed the amplitude of wave 5 attenuated 5%–10% (D).

**Figure 3.**
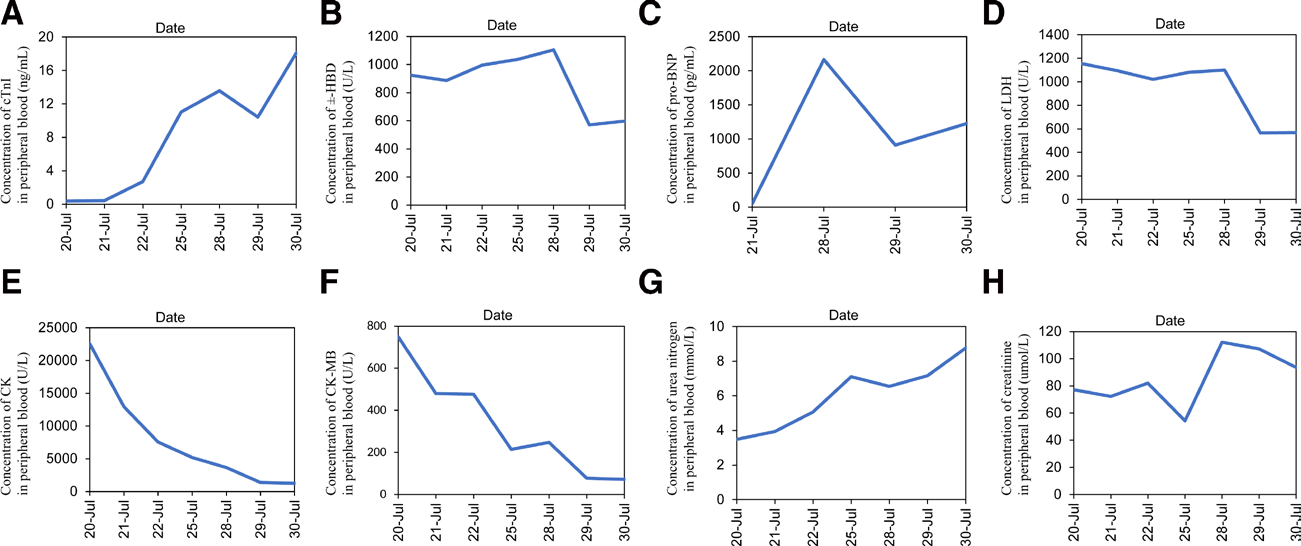
Changes in serum indicators during the course of the disease. Changes in cTnI, (A) α-HBD, (B) pro-BNP, (C) LDH, (D) CK, (E) CK-MB, (F) urea nitrogen, (G) and creatinine, and (H). Take the value of the examination at the onset as the base. CK = creatine kinase, CK-MB = creatine kinase-MB form, cTnI = troponin I, LDH = lactate dehydrogenase, α-HBD = alpha-hydroxybutyrate dehydrogenase, pro-BNP = pro-brain natriuretic peptide.

She was admitted to hospital with abdominal pain, intermittent chest tightness and dizziness 14 days after receiving therapy. The patient did not report fatigue and droopy eyelids until 2 days after admission. The patient’s vital signs were stable, with a temperature of 36.5°C, heart rate of 68 beats per minute, a respiratory rate of 20 breaths per minute, and blood pressure 130/89 mm Hg. Physical examination revealed mild tenderness in epigastrium. However, no abnormalities were found in pulmonary, cardiac and craniocerebral examinations. The patient has not experienced irAEs before. Her laboratory tests revealed elevated serum troponin I (cTnI) (0.3625 ng/mL, normal < 0.03 ng/mL), lactate dehydrogenase (1155.62 U/L, normal < 250 U/L), creatine kinase (22535.71 U/L, normal < 200 U/L), creatine kinase-MB form (748.92 U/L, normal < 24 U/L), and alpha-hydroxybutyrate dehydrogenase (α-HBD) (924.98 U/L, normal < 182 U/L). Electrocardiogram (ECG) reported the prolongation of QTc interval (Fig. 2A). Echocardiography showed small amount of pericardial effusion, a left ventricular ejection fraction of 71% and no abnormalities in cardiac structure, movement, blood flow, and ventricular systolic-diastolic function (Fig. 1E). Coronary artery CT angiography revealed localized noncalcified plaque in the middle of the left anterior descending artery and mild stenosis of the lumen (Fig. 1B). Enhanced CT scanning of the whole abdomen showed no abnormal signs in the parenchyma organs. Combining the results of the above examinations, the acute myocardial infarction, heart failure, cardiomyopathy, and aortic dissection was first excluded. The uremic cardiomyopathy was then excluded depending on the normal urine output, the abdominal CT scanning, and the examination of urea nitrogen (Fig. 3G) and creatinine (Fig. 3H). We next reviewed the medical history of that the patient did not have a history of infection recently and did not use drugs or treatment with myocardial damage except for sintilimab. The viral myocarditis was temporarily excluded. Considering the correlation between the use of drug and onset time of clinical symptoms, ICI-mediated myocarditis and MG was highly considered. Therefore, we proposed that cardiovascular magnetic resonance or positron emission tomography/CT or endocardial myocardial biopsy (EMB), electromyogram (EMG) and MG related antibodies need to be completed. But the patient and her families rejected the cardiovascular magnetic resonance, positron emission tomography/CT and EMB. The EMG showed that the amplitude of wave 5 caused by low-frequency re-frequency electrical stimulation on bilateral facial nerves attenuated 5% to 10% (Fig. 2D) and the antibody against acetylcholine receptor was positive. Based on the above information, the most likely diagnosis was myocarditis overlapping MG secondary to sintilimab. Therefore, the patient was treated with glucocorticoids (120 mg/day, IV, methylprednisolone) within 24 hours of admission. At the same time, the symptomatic and supportive treatments such as pain relief and stomach protection were initiated.

Five days later, the laboratory tests showed decreased lactate dehydrogenase (1081.4 U/L), creatine kinase (5191.2 U/L), and creatine kinase-MB form (214.67 U/L), but further increased cTnI (11.033ng/mL) and α-HBD (1037.6 U/L). The ECG reported complete right bundle branch block, the prolongation of QTc interval and the change of T wave (Fig. 2B). The multi-disciplinary treatment was carried out and the methylprednisolone (120 mg/day, IV) was advised to be continued. Two days later, the patient experienced tachy ventricular arrhythmia (Fig. 2C) and cardiogenic shock and was transferred to intensive care unit after electrical cardioversion, tracheal intubation and cardiopulmonary resuscitation. Intravenous immunoglobulin therapy at 25 g/day was given and methylprednisolone was reduced to 40 mg/day for the next 3 days. Intravenous esmolol and lidocaine were used for correcting arrhythmias. Ventilator positive pressure ventilation was used for respiratory support. The laboratory tests revealed continuously increasing cTnI (13.5767 ng/mL) and α-HBD (1104.93 U/L), and abnormal pro-brain natriuretic peptide (pro-BNP) (2164.4 pg/mL). The ECG monitoring showed ventricular arrhythmia storms. Therefore, she was administrated with plasmapheresis. After plasmapheresis, there was a temporarily decreasing in cTnI (10.4138 ng/mL) and pro-BNP (910.4 pg/mL), but no improving in ventricular arrhythmia storms. One day later, the cTnI (18.0849 ng/mL) and pro-BNP (1227.1 pg/mL) rapidly increased again, and ventricular tachycardia and ventricular fibrillation recurred. Amiodarone, the cardioversion and defibrillation were invalid for the ventricular arrhythmia storms. Then the patient’s condition progressed to cardiac failure, and her family members rejected the extracorporeal life support (ECLS), and she eventually died on July 31, 2022. Her families refused the autopsy. The changes in serum indicators (Fig. 3) during the treatment process of the disease were regularly reviewed. The outlines of the patients’ diagnosis and treatment can refer to Figure 1A.

All procedures performed in studies involving human participants were in accordance with the ethical standards of the institutional and/or national research committee(s) and with the Declaration of Helsinki (as revised in 2013). Written informed consent for publication was obtained from the patient’s family.

## 3. Discussion

In recent years, ICIs have been a promising therapy for malignancies.^[8]^ Anti-PD-1/PD-L1 monoclonal antibodies have been extensively studied in different type of cancers.^[3]^ The common side effects are reported as pneumonitis, hepatitis, endocrinopathies, dermatologic toxicity, gastrointestinal toxicity, and myositis.^[9]^ Recently, the rare irAEs such as cardiovascular toxicity has been reported frequently.^[9,10]^ The incidence of the ICI-associated myocarditis ranges from 0.09% to 1.14%, but the fatality rate is as high as 50% with monotherapy and 67% with combination immunotherapy.^[10]^

The case presented here is a middle-aged woman with unresectable thymoma. She was treated with sintilimab together with chemotherapy with the consent of her families and herself. She was admitted to hospital because of abdominal pain, intermittent chest tightness and dizziness after the treatment for 2 weeks. Experimental test showed significant elevated myocardial enzyme indicators. These were no typical signs indicating acute coronary syndrome in ECG and no systolic dysfunction of LV in cardiac ultrasonography with a 71% ventricular ejection fraction. Subsequent coronary artery CT angiography did not indicate pathogenic stenosis. To diagnose the ICI-induced myocarditis, we first exclude the fatal emergencies such as acute myocardial infarction, pulmonary embolism and aortic dissection. Acute chest pain and tightness may be caused by the acute myocardial infarction, which the coronary artery CT angiography may make a definitive diagnosis. Acute pulmonary embolism was often accompanied by sudden chest pain and gasp. The blood D-dimer increased significantly and the ECG usually indicated right ventricular injury. Aortic dissection presented as tear-like pain in the chest and back at the beginning, the location of the pain is consistent with the extent of the dissection and the blood pressure of both extremities was asymmetry. The symptom of the patients and examination results did not support these emergencies. Other differential diagnosis of myocarditis includes heart failure, cardiomyopathy, uremic cardiomyopathy and viral myocarditis. The patient mainly complained about intermittent chest tightness without cough, sputum or oliguria. And the echocardiography revealed a small amount of pericardial effusion and no abnormalities in cardiac structure and function. Therefore, we excluded the heart failure and cardiomyopathy. The uremic cardiomyopathy may exhibit kidney atrophy and cortex thinning in the CT scanning, and the serological tests may reveal the abnormal urea nitrogen and creatinine, so the uremic cardiomyopathy was excluded. Due to the lack of incentives and symptoms for infection, and the performance in ECG and echocardiography, viral myocarditis has been temporarily excluded. Considering the patient’s medical history, the onset time of the symptoms, the imaging tests and the serological findings, we sought that the ICI-induced myocarditis could be diagnosed. Subsequently, the patients presented with symptoms of fatigue and ptosis. The diseases that cause oculomotor nerve palsy and blepharospasm should be identified. Combining the ICI-usage, the onset time of ptosis, the EMG results and the positive antibody against acetylcholine receptor, the ICI associated MG was highly suspected. Then the patient was given immunosuppressive therapy with 2 mg/kg/day methylprednisolone while monitoring myocardial enzymes.

Understanding the pathogenesis of the ICI-induced myocarditis can better serve the clinical treatment. But the pathogenesis of the ICI-induced myocarditis is still unclear. One possible mechanism is that cardiac myocytes may share targeted antigens with the tumor, therefore becoming targets of activated T lymphocytes resulting in lymphocytic infiltration of the myocardium.^[11]^ Some of the reported cases containing EMB revealed that the pathological findings of ICI-induced myocarditis are identical to lymphocytic myocarditis.^[12]^ The lymphocytic infiltration within the myocardium primarily comprises a population of PD-1-positive, CD8-positive, and granzyme-B-positve T lymphocytes.^[13]^ The other possible mechanism is the absence or additional blockade of the PD-L1-PD-1 axis breaks peripheral immune tolerance resulting in heart-specific autoimmune-related myocarditis. In mouse models of T-cell mediated myocarditis, PD-1 was found to be upregulated and play an important role in myocardial immune responses and protect against inflammation and myocyte damage.^[14]^ This was consistent with the findings observed in the injured myocardium in patients. The deletion of PD-1 caused the production of autoantibodies and reduction of regulatory T cells, which eventually resulted in cardiomyopathy.^[14]^ In general, the ICIs therapy enhances the release of tumor-specific T cells, but weakens the immune tolerance, finally leading to the activation of self-reactive effector cells and heart tissue damage.

Based on the histopathologic of lymphocytic myocarditis, the main strategy to deal with ICI-induced cardiotoxicity is immunosuppressive therapy.^[9]^ The intravenous glucocorticoid should be given immediately. For the hemodynamically stable patients, prednisone at initial dose of 1 to 2 mg/kg/day or equivalent dose of methylprednisolone should be recommended. For the patients with grade 3 to 4 myocarditis, especially with fulminant myocarditis or malignant arrhythmia, the 1000 mg/day intravenous methylprednisolone should be initiated and maintained for 3 to 5 days.^[9]^ Shujing Liang et al^[15]^ reported a case of sintilimab-induced myocarditis in relapsed chordoma, who was rescued by using methylprednisolone (160 mg, q8 hours) for 5 days, which is consistent with our processing. Another similar case was reported by Lee S Nguyen et al^[12]^ The patient was diagnosed with ICI-reduced fulminant myocarditis and treated with 1000 mg/day methylprednisolone at the beginning.^[9]^ For these patients who do not well respond to hormone shock, the other recommendable immunomodulatory drugs contain the following categories. The chemical drugs such as tacrolimus, azathioprine, methotrexate and mycophenolate mofetil, the biological agents such as infliximab, rituximab, ruxolitinib, abatacept, and tocilizumab and the immunoglobulin such as antithymocyte globulin.^[9,12]^ If the patient’s condition continues to deteriorate, the plasmapheresis, lymphocyte depletion and ECLS could be proposed.^[12]^ The condition of the patient reported by Lee S Nguyen deteriorated to cardiogenic shock and persistent ventricular tachycardia. He was transferred to ICU for ECLS and was successfully rescued with the use of personalized-dose-adjusted abatacept and ruxolitinib,^[12]^ which is lack in our case and is worth of reference. Except for the immunosuppressive therapy, the common respiratory and hemodynamic support treatment should be employed for patients according to the guidelines.^[9]^ Comparing our reported case with others, the lessons learned from it are: using adequate methylprednisolone as soon as possible and developing individualized treatments with diverse immunosuppression and novel drugs the within the allowance of medical conditions when the patient do not well respond to hormone shock.

## 4. Conclusion

The ICIs therapy in thymoma showed promising results, but the life-threatening irAEs is the biggest risk. A major challenge to consider while treating thymoma patients is how to screen the immunotherapy benefit groups or exclude high-risk groups of adverse events, and how to make immunotherapy a safe and effective option. Deeply study the mechanism of the ICI-induced toxicity in thymoma patients may provide valuable insight into the interaction between the immune system with the tumor cells and make the immunotherapy more meaningful in thymoma treatment.

## 5. Patient perspective

From the perspective of the patient, the symptoms at the beginning were tolerable, but gradually aggravation until death. Her experience was frightening. The patient and her families may not trust our team so much that they rejected some of the necessary tests and ECLS. The expensive medical expenses in ECLS and some novel drugs were also the most concern for the patient and her families.

## Author contributions

**Conceptualization:** Chen Wang, Xiaohong Liao

**Data curation:** Bingdi Zhong, Xiaohong Liao.

**Formal analysis:** Jing He.

**Methodology:** Bingdi Zhong

**Supervision:** Chen Wang.

**Writing – original draft:** Bingdi Zhong, Xiaohong Liao.

**Writing-review & editing:** Chen Wang, Jing He.
